# *Vicia*–Micronucleus Test Application for Saline Irrigation Water Risk Assessment

**DOI:** 10.3390/plants11030462

**Published:** 2022-02-08

**Authors:** Dalila Souguir, Ronny Berndtsson, Sourour Mzahma, Hanen Filali, Mohamed Hachicha

**Affiliations:** 1 Laboratory of Non-Conventional Water Valorization, National Research Institute of Rural Engineering, Water, and Forestry, University of Carthage, LR16INRGREF02, 10 Rue Hédi Karray, Manzeh 4, Ariana 2080, Tunisia; sourourmzahma@gmail.com (S.M.); hanenfilali.26@gmail.com (H.F.); hachicha.mohamed@iresa.agrinet.tn (M.H.); 2 Centre for Advanced Middle Eastern Studies, Division of Water Resources Engineering, Lund University, SE-22100 Lund, Sweden; 3 Faculty of Sciences of Bizerte, University of Carthage, Jarzouna 7021, Tunisia

**Keywords:** plant aberration, cell division, plant growth, micronucleus, salinity, *Vicia faba*

## Abstract

In view of climate change, increasing soil salinity is expected worldwide. It is therefore important to improve prediction ability of plant salinity effects. For this purpose, brackish/saline irrigation water from two areas in central and coastal Tunisia was sampled. The water samples were classified as C3 (EC: 2.01–2.24 dS m^−1^) and C4 (EC: 3.46–7.00 dS m^−1^), indicating that the water was questionable and not suitable for irrigation, respectively. The water samples were tested for their genotoxic potential and growth effects on *Vicia faba* seedlings. Results showed a decrease in mitotic index (MI) and, consequently, growth parameters concomitant to the appearance of micronucleus (MCN) and chromosome aberrations when the water salinity increased. Salt ion concentration had striking influence on genome stability and growth parameters. Pearson correlation underlined the negative connection between most ions in the water inappropriate for irrigation (C4) and MI as well as growth parameters. MI was strongly influenced by Mg^2+^, Na^+^, Cl^−^, and to a less degree Ca^2+^, K^+^, and SO_4_^2−^. Growth parameters were moderately to weakly affected by K^+^ and Ca^2+^, respectively. Re-garding MCN, a very strong positive correlation was found for MCN and K^+^. Despite its short-term application, the *Vicia*-MCN Test showed a real ability to predict toxicity induced by salt ions confirming that is has a relevant role in hazard identification and risk assessment of salinity effects.

## 1. Introduction

During their life cycle, plants face a range of environmental stress and must adjust their growth to the surrounding conditions. The quality of water around their roots is an important component that may affect plant development. Salinity of irrigation water is a problem in irrigated areas. Several different criteria are used to classify the suitability of water for irrigation, including electrical conductivity (EC), sodium adsorption ratio (SAR), and presence of moderate to high concentrations of specific ions [1,2]. Salinity limits plant growth and production due to water deficit, ion toxicity, and oxidative burst. The detrimental effects of salinity involve disruption of the morpho-logical, biochemical, cellular, and molecular functions [3,4,5,6]. Roots are the first barrier to salt ions that may prevent their passage into the plant. However, when the filtration of salts through the roots is defective, plant growth is dependent on the ability of the plant to keep salt from interfering with its metabolic processes by modulating gene expression and protein activity as well as compartmenting toxic ions. Changes in the root architecture, wall composition, and transport processes are among the main modifications occurring in the roots when salinity is high [7,8].

Salt stress affects all growth stages of plants. Germination and seedling growth, the most sensitive stages, are affected partly due to inhibition of the cell cycle and cell growth working in coordination to ensure growth. The cell cycle, responsible for the increase of cell number, comprises a series of events that allow doubling of cellular components and segregation into daughter cells while cell growth, also called cell expansion, refers to the increase in cell size [8,9]. The cell cycle consists of four phases including the M phase where the cells divide, the S phase where the DNA is replicated and prepares for division, and the G1/G2 phase in between [9,10]. Salinity has been found to decrease cell division rate and, consequently, cell number and to induce micronuclei and chromosome/nucleus abnormalities responsible for plant size reduction [11]. The Micronucleus Test (MCN Test) is used to assess the toxic potential of geno-toxic agents and it allows for control of mitosis [12,13,14,15]. From a practical point of view, the MCN Test is fast since an exposure of only 48 h is required, not expensive compared to more advanced laboratory tests, requires only a microscope (and camera), and allows for assessment of genotoxic parameters (cells in division, MCN, and chromosome/nucleus aberrations) in the same slide [15].

MCN is an extranuclear entity of the damaged part of chromosomes and its origin is attributed to both structural and numerical alterations of chromosomes and, probably, to elimination of excessive genetic material from the main nucleus [16,17,18]. Cell growth, leading to increase of the individual cell’s volume, is strongly influenced by intracellular turgor pressure, cell wall rigidity and flexibility allowing for various cell sizes and shapes [19]. Modification of the mechanical properties of the cell wall is considered as a major growth-limiting factor during exposure to salinity. It is linked to changes of cell wall capacity to expand [20]. In this regard, a strong relationship has been found between cell wall stiffening and reduction of the epidermal cell size of maize during an osmotic stress-phase induced by salt treatment [3].

Faba bean (*Vicia faba*) is a leguminous vegetable that is rich in protein, widespread in Tunisia and other semi-arid areas, and known as a salt-sensitive species. The plant is easy and quick to grow, inexpensive, and an excellent genetic model for environmental monitoring and risk assessment. *V. faba* has a small chromosome number (2n = 12) with large shape easily observed with microscope [13]. In this regard, the plant enables assessment of genomic integrity through the following endpoints: mitotic index (MI), micronucleus induction (MCN), and chromosomal and nuclear aberrations. The MCN Test is innovative since it is efficient and rapid and can reduce cost for water and soil analyses. Therefore, the first objective of the present study was to improve the general understanding of relationships between salinity, ionic composition, genotoxicity, and growth parameters of *V. faba* seedlings. Secondly, we wanted to determine whether the cell division of *V. faba* seedlings during exposure to saline irrigation water can be used for salinity risk assessment and comparison of different salinity exposures. We investigated roots of *V. faba* because of their role as a first barrier in contact with salt ions. In addition, we studied meristematic cells since these are considered salt-sensitive and they harbor mitotic activity that is indispensable for root growth.

## 2. Results

### 2.1. Irrigation Water Assessment

The pH of the experimental irrigation water samples was neutral to basic varying between 7.01 to 8.07 (Table 1). The electrical conductivity (EC) varied from 2.01 to 7.00 dS m^−1^. In total, 26 C3 class samples (doubtful) and 24 C4 class samples (unsuitable) according to the US Salinity Staff classification [1] were used in the experiments. For C3, EC ranged from 2.01 to 2.24 dS m^−1^ with an average of 2.10 dS m^−1^ (CV = 4.5%). The C4 samples included an EC ranging between 3.46 and 7.00 dS m^−1^ with an average of 5.13 dS m^−1^ (CV = 23.3%).

Sodium (Na^+^) was the predominant cation in both C3 and C4 samples whereas chloride (Cl^−^) was the predominant anion. Na^+^ varied between 6.23 and 19.86 me L^−1^ in C3 with an average of 14.86 me L^−1^ and between 39.00 and 69.00 me L^−1^ in C4 with an average of 51.58 me L^−1^. Cl^−^ values were close to those of Na^+^ with an average of 14.19 me L^−1^ and 54.04 me L^−1^ in C3 and C4, respectively.

High sodium adsorption ratio (SAR) can lead to decrease in soil permeability and crop production. Average SAR was 5.99 (3.46–7.65) for C3 reflecting a low sodium hazard of water samples. For C4, the SAR was equal to 13.25 (10.57–15.51) indicating a medium sodium hazard. Considering the joint effect of EC and SAR, the sampled groundwater can probably be used for irrigation together with an adapted salinity management and salt leaching.

### 2.2. Genotoxicity Assessment

**Figure 1:**
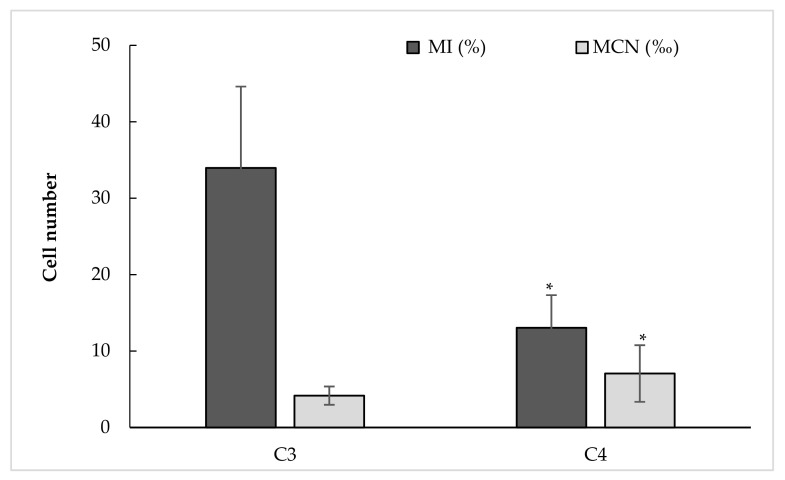
Genotoxicity represented by mitotic index and cells with micronucleus per 100 and 1000 counted cells, respectively, in *Vicia* root meristematic zone after 48 h of exposure to C3 (EC: 2.01–2.24 dS m^−1^) and C4 (EC: 3.46–7.00 dS m^−1^) irrigation water. MI = mitotic index, MCN = micronucleus. Values are mean ± SD (n = 26 for C3 and 24 for C4). * indicates significant difference between C3 and C4 at *p* < 0.05.

All water samples were subject to a genotoxicity assessment. Dividing cells represented by mitotic index (MI) and cells with a micronucleus (MCN) per 100 and 1000 cells, respectively, are shown in Figure 1. The percentage of dividing cells exposed to C3 irrigation water oscillated between 16 and 53.0% with an average of 34.0% and medium CV of 31.3%. The increase of salt in the irrigation water was followed by a decrease in the number of dividing cells. This was clear, through a significant decline (61.6% reduction) of MI in *Vicia* roots receiving water with unsuitable quality of irrigation water (C4). In this group, the MI range was 6.3–22.0% with an average of 12.7% and a lowest value of 6.3% for water with the highest EC (about 7 dS m^−1^). 

**Figure 2:**
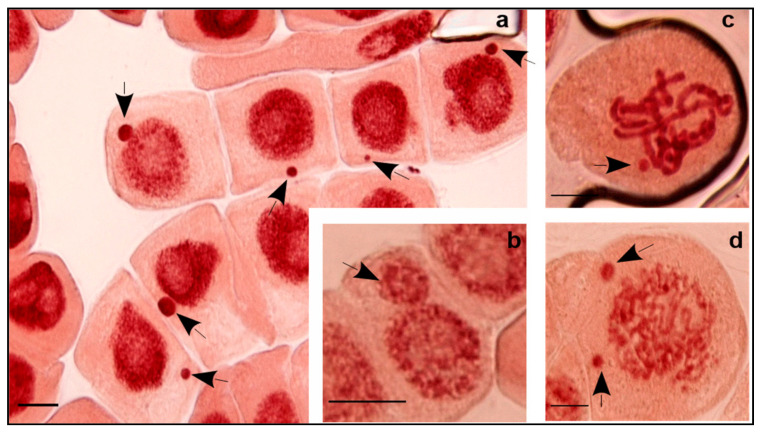
*Vicia* cells with different size and number of micronucleus (MCN) under exposure to C4 water samples. The size of a MCN was compared to the main nucleus. (**a**) Cells with a small or a medium MCN (EC = 4 dS m^−1^); (**b**) Cell with a large MCN (EC = 6 dS m^−1^); (**c**) Cell in division with a MCN (EC = 4 dS m^−1^); (**d**) Cell in division with two MCNs (EC = 6 dS m^−1^). Arrow indicates MCN. Magnification: 1000×; Bar = 20 µm.

Cell division progress was accompanied by appearance of cells supporting MCN. Different sizes and numbers of this entity were found when screening microscopic preparations (Figure 2). Observed micronuclei were small, medium, and large compared to the main nucleus. A cell in either interphase or in division may include one and more than one MCN. Only interphasic cells with one MCN were considered in this study. The average was 4.2‰ (2.4–7.5‰) for C3 and, significantly, much higher at 7.2‰ for C4 (69.5% increase), previously exhibiting a lower rate of cell division (Figure 1).

**Figure 3:**
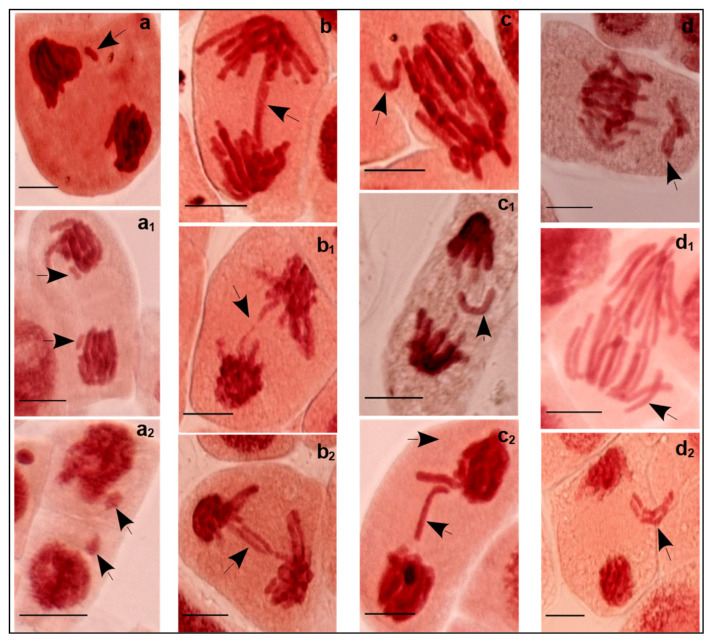
Structural and numerical anomalies found in *V. faba* cells in division exposed to C3 and C4 irrigation water samples. Structural aberrations include changes in structure of chromosomes: (**a**) Cell in anaphase with a chromosomal fragment (EC = 6 dS m^−1^); (**a1**) Cell in anaphase with two chromosomal fragments (EC = 6 dS m^−1^); (**a2**) Cell in telophase with two chromosomal fragments (EC = 7 dS m^−1^); (**b**) Cell in anaphase with a bridge (EC = 4 dS m^−1^); (**b1**) Cell in anaphase with a broken bridge (EC = 6 dS m^−1^); (**b2**) Cell in anaphase with two bridges (EC = 6 dS m^−1^). Numerical aberrations include changes in number of chromosomes: (**c**) Cell in anaphase with a lagging chromatid (EC = 6 dS m^−1^); (**c1**) Cell in anaphase with a lagging chromatid (EC = 2.2 dS m^−1^); (**c2**) Cell in anaphase with two lagging chromatids (EC = 6 dS m^−1^); (**d**) Cell at the beginning of anaphase with lagging chromosome (EC = 2.2 dS m^−1^); (**d1**) Cell in anaphase with lagging chromosome (EC = 4 dS m^−1^); (**d2**) Cell at the end of anaphase with lagging chromosome (EC = 6 dS m^−1^). Arrow indicates aberration. Magnification: 1000×; Bar = 20 µm.

Concomitant to MCN appearance, anomalies in chromosomes/chromatids are especially involved in anaphase and telophase (Figure 3). Structural anomalies are presented by fragments and anaphase bridges. Numerous anomalies are predominately a consequence of segregation defects of chromosomes. Lagging chromosomes/chromatids are lost in the cell.

### 2.3. Growth Parameters

Monitored growth parameters concerned root length (RL), and fresh and dry mass (FM and DM), respectively (Table 2). In general, all parameters showed decrease when C4 samples were compared to those of C3. The decrease of root elongation was equal to 11.1%. For C4, roots were shortest (2.20 cm) in the case of highest EC (7 dS m^−1^) and longest (3.80 cm) with lowest EC (about 3.72 dS m^−1^). Similarly, fresh and dry matter that were 0.31 and 0.027 g, respectively, decreased by 10.5% and 9.6%, respectively, in C4. 

### 2.4. EC Effect on Genotoxic and Growth Parameters

Table 3 presents correlation between EC and genotoxic and growth parameters for the C3 and C4 water. Correlation coefficients are considered very strong when r ranges from 0.90 to 1.00, strong from 0.70 to 0.90, moderate from 0.50–0.70, weak from 0.20 to 0.50, and very weak when the r is below 0.20. For C3, no remarkable effects of EC were noticed on MI (r = 0.163, *p* < 0.05), MCN (r = 0.258, *p* < 0.05), or growth parameters (RL (r = 0.244, *p* < 0.05), FM (r = 0.197, *p* < 0.05) and DM (r = 0.199, *p* < 0.05)). Significant correlation was only observed between MI and RL (r = 0.518, *p* < 0.01).

For C4, EC was significantly correlated with genotoxic parameters with negative relationship between EC-IM (r = −0.764, *p* < 0.01) and, in contrast, positive relationship between EC-MCN (r = 0.561, *p* < 0.01) indicating that increase of EC is followed by a decrease of MI and increase of MCN. MI and MCN presented an inverse relationship (r = −0.496, *p* < 0.05). Similarly, *Vicia* root elongation displayed a significant negative cor-relation with MCN (r = −0.519, *p* < 0.01), albeit weak and non-significant in case of FM and DM with r equal to −0.347 and −0.362 at *p* < 0.05 for MCN-FM and MCN-DM, re-spectively. 

### 2.5. Ion Effects on Genotoxic and Growth Parameters 

Relationships between cations (Na^+^, Ca^2+^, Mg^2+^, and K^+^) as well as anions (Cl^−^, SO_4_^2−^ and HCO_3_^−^) present in water samples and different variables (MI, MCN, RL, FM, and DM) are shown in Table 4. No significant influence of the studied ions on genotoxic parameters was noticed for C3 water with low EC. However, significant negative correlation was observed between MI/MCN and cations/anions in samples with higher salinity (C4). Strong negative correlation typified MI-Mg^2+^ (r = −0.827, *p* < 0.01), MI-Na^+^ (r = −0.771, *p* < 0.01), and MI-Cl^−^ (r = −0.805, *p* < 0.01), and to a less degree MI-Ca^2+^ (r = −0.465, *p* < 0.05), MI-K^+^ (r = −0.417, *p* < 0.05), and MI-SO_4_^2−^ (r = −0.518, *p* < 0.01). Regarding MCN, a very strong positive correlation was found between MCN-K^+^ (r = 0.915, *p* < 0.01) while weak to moderate correlation characterized the other elements (Na^+^, Ca^2+^, Mg^2+^, Cl^−^ =, and SO_4_^2−^). Ions had no remarkable effect on growth parameters at low salinity except for K^+^. However, with the increase in salinity, RL, FM, and DM were influenced by K^+^ and Ca^2+^. The HCO_3_^−^ content in C3 and C4 did not display any significant effect on growth parameters.

## 3. Discussion

Water salinity is of main concern for agriculture in arid and semi-arid areas. Irrigation with brackish water is often the only alternative for farmers in these areas. The C3 water had an average of salinity of 2.10 dS m^−1^ (oscillating between 2.01 and 2.24 dS m^−1^), which is viewed as rather good quality water in semi-arid Tunisia. The C4 water had salinity content above 3.46 dS m^−1^ that reached 7 dS m^−1^. Na^+^ and Cl^−^ were the most abundant ions in this water. The effect of both salinity groups was evaluated regarding genotoxicity and root growth of *V. faba* germinated seedlings. 

### 3.1. Effect of Salinity on Cell Cycle and Growth 

Visual effects of high salinity were generally low root and shoot growth and a significant production loss. Being the primary receptor of salt stress and the site of cell cycle and growth, roots act as a barrier against the entry of toxic ions. Cell cycle and cell growth increase cell number and cell volume, respectively, which enables the plant to increase in size. The number of cells in the tissue increases through the mitotic cycle while the volume of individual cells increases through cytoplasmic growth and turgor-driven cell expansion [8,9,19,21]. As an indicator of cell cycle-linked events, we investigated the mitotic cycle progression by counting dividing cells (MI%). Its resilience was assessed by counting micronucleus (MCN‰) and photographing chromosomal abnormalities. Roots affected by C3 water (varying between EC of 2.01 and 2.24 dS m^−1^) had an MI average of 34.0% (16–53%), a low MCN equal to 4.2‰ (2.0–7.5‰), mean root length of 3.38 cm, and FM and DM equal to 0.31 and 0.027 g, respectively. No effects of EC on mitotic cycle progression, MCN formation, as well as growth parameters were observed for C3. Instead, significant positive correlation was found between dividing cell rate and root length (0.518, *p* < 0.01) 

The increase of salinity (C4) triggered a significant decrease in the number of cell divisions 12.7% (6.3–22.0%) and, inversely, an increase of MCN formation of 7.2‰ (2–19.5‰) compared to C3. Results showed that high EC resulted in negative correlation with MI (r = −0.764, *p* < 0.01) while positive correlation was found between EC and MCN (r = 0.561, *p* < 0.01). This is interpreted as salinity that damages the cell cycle through a decrease in dividing cell numbers and alteration of chromosome integrity which, consequently, generate MCN. Only cells with normal division phases were counted, which may, in part, explain this decrease. Abundant defective cells in different mitotic phases containing chromosome/chromatid aberrations were excluded. The defective cells generally induced MCN that are formed from acentric chromosome/chromatid fragments or the whole chromosome/chromatid that lag in anaphase and fail to be incorporated in the daughter nucleus. Probably also, this process is affected by nuclear buds attached to the nucleus by a thin nucleoplasm connection [22]. 

Cell division affects the root length, as an important indicator of salt stress sensitivity/tolerance. In our case, reduction in root growth was observed for the high-saline water (C4). The largest root length for C4 (3.80 cm) was observed for 3.72 dS m^−1^ and the smallest (2.20 cm) for 7 dS m^−1^. FM and DM (fresh and dry matter, respectively) decreased, in turn, by 10.5 and 9.6%, respectively, compared to C3 water, despite the short exposure to saltwater (only 48 h). Such reduction in root growth is, in part, the result of decrease in dividing cell number (MI decrease) and the appearance of abnormal cells with MCN. This was confirmed by the negative correlation between MCN and RL (r = −0.519, *p* < 0.01), albeit weak and non-significant in case of FM and DM. The effect of salinity on cell production was studied by West et al. who showed that growth decrease of the primary roots of Arabidopsis under salt stress was induced by decrease in cell number and smaller mature cell length [7]. They emphasized the inhibitor effect of salt on the cyclin dependent kinases’ (CDKs) activities. CDKs are regulatory proteins that control cell division and modulate transcription in response to harmful conditions, by complexing with the cyclins [23]. In fact, the progression of the cell cycle, which is formed by G1, S, G2, and M (mitosis) phases, is controlled by checkpoints. Cell cycle checkpoints ensure control of cell size and accurate replication and integrity of the chromosomes by preventing cells with damaged or incompletely replicated DNA from entering mitosis and promote the appropriate segregation at mitosis through a control of mitotic spindle [24]. Checkpoints, CDKs, as well as cyclin activity defects with reduced root growth under salt stress have been well documented [7,9,23] and in part explain the various clastogenic and aneugenic aberrations found under salinity conditions. Chromosomal fragments and bridges are considered as structural abnormalities that may promote smaller MCN size (clastogenic action of salt). Laggings (chromosomes or chromatids) are numerical abnormalities that may induce larger MCN size (aneugenic effect of salt).

The suppression of plant hormone signaling pathways resulting in lower cell cycle activity also results in decreased root elongation. Plant hormones include abscisic acid, auxin, cytokinin, brassinosteroid, gibberellin, and ethylene. These are considered as essential molecules for root growth processes, promoting cell division, cell expansion and elongation, and cell differentiation [7,25,26,27]. Harmful effects by salt have been attributed to the inhibition of calcium signaling pathways and reactive oxygen species generation causing oxidative damages to nucleic acid bases, by modifying bases and favoring single or double strand breaks in DNA, altering cytosine methylation, and activating programmed cell death (e.g., [28,29,30]).

### 3.2. Salt Ion Interference with Genome Stability and Growth

Detrimental effects of salt may lead to toxicity due to specific ions. Specific ion toxicity is usually associated with excessive intake of Na^+^, Cl^−^, or other ions, which, once in the plant, disrupt the ion homeostasis mechanisms and damage plant functions. Cell division integrity and root elongation are not excluded from such damage. In this regard, the relationship between, on one hand, genotoxicity, and root growth parameters, and, on the other hand, cations (Na^+^, Ca^2+^, Mg^2+^, and K^+^) as well as anions (Cl^−^, SO_4_^2−^, and HCO_3_^−^), was investigated. Mitotic division was negatively influenced by abundant or lower concentration of salt ions in the medium, except for HCO_3_^−^. Correlation was strong for Mg^2+^ (r = −0.827, *p* < 0.01), Na^+^ (r = −0.771, *p* < 0.01), and Cl^−^ (r = −0.805, *p* < 0.01) and moderate for Ca^2+^ (r = −0.465, *p* < 0.05), K^+^ (r = −0.417, *p* < 0.05), and SO_4_^2−^ (r = −0.518, *p* < 0.01). The formation of micronuclei entities was related strongly to K^+^ (r = 0.915, *p* < 0.05) and moderately or weakly to other ions. Growth pa-rameters such as root length, in turn, were strongly negatively correlated to the main cations K^+^ and Ca^2+^ found at high concentrations in C4 water. 

Negative correlation was found between MI and salt ions, and positive correlation for MCN and salt ions. This shows that cations/anions exert mitodepressive effects at higher salt concentrations (C4 compared to C3). It is also a signal of mitosis sensitivity to the ion level in the medium which may, once in the cytosol, interfere with the cell cycle regulatory processes. Among cations that display interference with cell division, Mg^2+^ is involved in the formation of mitotic spindle through polymerization and de-polymerization of microtubules (e.g., [31]). Abraham and Nair studied cytological changes produced in root meristems of *V. faba* after exposure to magnesium sulfate (MgSO_4_) [32]. In addition to its ability to induce micronuclei, chromosome breakage, chromosome clumping, achromatic lesions, and lagging chromosomes, Mg^2+^ at high concentrations has been found to induce spindle abnormalities leading to the formation of polyploidy and aneuploidy [32]. Na^+^ and Cl^−^ have been shown to induce genome disorder. Boyko et al. observed drastic increase of the recombination rates in the presence of Cl^−^ ions, leading to increased numbers of double strand breaks during DNA replication. Na^+^ ions, however, had no remarkable effect on the frequency of genomic rearrangements [33]. 

In addition to the strong correlation between MI and Mg^2+^, Na^+^, and Cl^−^, moderate effects were observed for Ca^2+^, K^+^, and SO_4_^2−^ for C4 water. SO_4_^2−^ is the primary source of sulfur vital for cell division and protein synthesis and its availability strongly influences plant growth and development as well as crop yield and quality. Short and long-term deprivation of SO_4_^2−^ or sulfur affects the cell cycle progress while effects of high concentrations of this ion have been less described (e.g., [34]).

K^+^ and Ca^2+^ are both needed to promote cell division and growth. Negative correlation was found between these two cations and MI as well as growth parameters for C4 water. The two cations help to maintain the selectivity and integrity of the cell membrane. K^+^ is the most abundant inorganic cation in plants, comprising up to 10% of plant dry matter and it is paramount for ensuring optimal plant growth. It is required for proper cell cycle progression during the transition from G1 to S phase, but high concentrations of K^+^ can cause gradual decrease in MI and various chromosome abnormalities [35]. K^+^ is also involved in cell elongation, and many other physiological plant functions (e.g., [36,37,38,39]). When high contents of Na^+^ occur, K^+^ uptake is disrupted in favor of Na^+^ entry. Plants attempt to maintain a high K^+^/Na^+^ ratio in the cytosol by regulating the activity of K^+^ and Na^+^ transporters and H pumps that generate the driving force for transport. However, due to the similar radii of K^+^ and Na^+^ in their hydrated forms, transport proteins are unable to discriminate between these two ions. Increase in the influx of Na^+^ under conditions of high external Na^+^, induces an alteration of the K^+^/Na^+^ ratio following the substitution of Na^+^ through K^+^ transporters or channels [38,40]. 

Ca^2+^ is needed for selective uptake and transport of K^+^ across membranes. Therefore, salt stress alleviation by Ca^2+^ is attributed to the role of this cation to increase K^+^ uptake and transport and prevent Na+ binding to the cell wall and plasma membrane [41]. Ca^2+^ appears to participate in the regulation of several events of cell division including nuclear envelope breakdown and reformation, chromatin fiber compaction, and chromosome segregation at anaphase and cytokinesis. It has been reported that, under abiotic stress, the increase of cytosolic concentrations of Ca^2+^ in S phase may be a signal of DNA damage and cell cycle regulation by modulating cyclin expression. Ca^2+^, at high exogenous concentrations, has been found to delay cell cycle progression and disrupt anaphase at spindle fiber and chromosome movement levels [42,43,44]. This cation is required for various structural functions in the cell wall and membranes as it can form intermolecular linkages [45] and constitute a second messenger in many biological systems [46,47,48]. 

## 4. Materials and Methods

### 4.1. Water Sampling and Analysis 

Water samples were collected during the wet (March) and a dry season (July) from shallow groundwater at Teboulba located in the Governorate of Monastir, Tunisian Sahel, and from deep groundwater at Merguellil located in the Governorate of Kairouan, Central Tunisia. Water samples were also obtained from concentration, through slow evaporation (at 55 °C), of irrigation water to predict the evolution of its quality under climatic change. After collecting the samples, EC (EC/TDS meter, AD 330, Adwa, Romania) and pH (pH/EC/TDS/Temperature meter, HI991300, Hanna, Romania) were measured and samples were then stored in a refrigerator for chemical analysis, genotoxic potential evaluation, and root growth parameter determination. 

Chemical analysis concerned sodium (Na+), calcium (Ca^2+^), magnesium (Mg^2+^), potassium (K^+^), chloride (Cl^−^), sulfate (SO_4_^2−^) and bicarbonate (HCO_3_^−^). The concentrations of Na^+^ and K^+^ were determined by flame emission spectroscopy (PFP7 Flame Photometer, Jenway, UK), Ca^2+^ and Mg^2+^ with complexometric titration method using a solution of Ethylenediaminetetraacetic acid (EDTA), Cl^−^ with the silver-nitrate (Ag-NO_3_) method, SO_4_^2−^ with the nephelometric method using barium chloride (BaCl_2_), and HCO_3_^−^ with titration with sulfuric acid (H_2_SO_4_). The sodium hazard (sodium adsorption ratio-SAR), evaluating the potential for infiltration problems, was calculated from the concentrations of Na^+^, Ca^2+^, and Mg^2+^. 

Based on salinity content, four classes of irrigation water have been proposed according to their EC [1]: low (C1), medium (C2), high (C3) and very high (C4) salinity hazard (Table 5). The classes C1 (EC: 0.10–0.25 dS m^−1^) and C2 (EC: 0.25–0.75 dS m^−1^) are considered safe and suitable for irrigation purposes. The class C3 (EC: 0.75–2.25 dS m^−1^) is doubtful and can be used based on specific management practices, while class C4 (EC > 2.25 dS m^−1^) is not suitable for irrigation under ordinary conditions.

According to the United States Salinity Laboratory Staff [1], four classes of sodium hazard, presented as sodium adsorption ratio (SAR), are distinguished with low to very high level (Table 6).

Guidelines for the interpretation of irrigation water quality according to the SAR and EC content are given in Table 7. In general, no problems for soil or crops are recog-nized when using water with values less than those shown for “no restriction on use” while increasing care in selection of crop and management is required under “slight and moderate restriction on use”. For water with severe restriction, a high level of management skill is essential for acceptable plant production. 

### 4.2. Genotoxicity Analysis and Growth Parameters

To evaluate the genotoxic potential of water samples, the Micronucleus Test (MCN Test) was applied on *V. faba* root tips according to Souguir et al. [49]. Dry seeds of *V. faba* (Tunisian variety Chahbi) were soaked for 24 h in distilled water. Seedlings were grown between layers of moist cotton for 3 days after removing a part of the seed coat to facilitate germination. The germinated seeds (with 2–3 cm of root length) were then transferred to plastic trays (30 × 21 cm^2^) which were filled with 400 mL of the irrigation water. After 48 h of exposure, roots were cut and placed overnight in a fixation solution containing ethanol and glacial acetic acid (3:1). Fixed roots were stored in the dark in 70% ethanol before being hydrolyzed with 1 N HCl. Microscopic preparations were stained with orcein and examined under a research Leica microscope (Leica DM2500, Wetzlar, Germany).

Cell cycle progression was monitored via mitotic index (MI) and cell cycle integrity was assessed via micronucleus and chromosome/chromatid aberrations appearance. The MI, which is the percentage of dividing cells (MI%), was determined by counting cells supporting one of the four basic mitotic phases: prophase, metaphase, anaphase, and telophase among a total of 100 cells. The MCN frequency reflected the number of interphase cells with micronucleus per 1000 scored cells (MCN‰). MCN is distinguishable by the main criteria of isolated nuclear structure separated from the main nucleus, smaller than the nucleus and with a staining intensity like that of the nucleus. At least three root meristems were considered per replica. For each water sample, three replicates were performed on at least an average of 900 and 9000 scored cells to determine MI and MCN frequency, respectively. Chromosome/chromatid aberrations occurring during division stages were photographed with a digital camera (Canon EOS 1100, Tokyo, Japan) attached to the Leica microscope with ×100 objective. 

At the end of the experimental period, root length (RL) of at least 9 plants was measured for each replicate and then harvested for fresh and dry mass determination. Fresh roots were weighed to determine fresh mass (FM). Dry mass (DM) was obtained after drying the roots in an oven at 70 °C until reaching a constant weight.

### 4.3. Statistical Analysis

Data were collected from experiments carried out with at least three replicates. Values relative to genotoxic parameters (MI and MCN) were presented as mean ± standard deviation (SD). To compare the effects of both water irrigation classes on genotoxicity and growth parameters, Student *t*-test at the 0.05 confidence level was performed. The Pearson correlation (r) was used to study relationships between varia-bles using the SPSS software (IBM SPSS Statistics, v20).

## 5. Conclusions

We investigated the relationship between water salinity (EC and ionic composition) and plant behavior (genotoxicity and growth parameters). High levels of salinity disrupted cell cycle integrity through an inhibition of mitosis accompanied by appearance of abnormal cells supporting micronucleus and chromosome/chromatid aberrations. Such cell cycle disturbance influences root growth through decrease in root elongation and fresh and dry matter content. Salt ion concentration strikingly influenced the genome stability and growth parameters. Negative correlation was found between most cations/anions, at high concentration in C4 water, and mitotic/growth parameters. The occurrence of micronuclei/aberrations proved the sensitivity of the *Vicia*-MCN Test to clastogenic/aneugenic effects of high levels of ions contained in the irrigation water. Despite the short-term application (48 h), MCN Test showed a good ability and rapidity to predict toxicity induced by salt ions that confirms its relevant role in hazard identification and risk assessment. *V. faba*, in turn, seems to be reliable for screening and monitoring irrigation water quality and possible soil and plant hazards. The plant salt-sensitivity makes it favorable to identify salt ions involved in DNA damages and abnormalities occurring during cell division.

Chemical analyses are often adopted to evaluate irrigation water and soil. Such analyses are expensive, especially for small farmers who, in many cases, need to control the quality of their irrigation water/soil. Including MCN Test among laboratory analyses performed for water/soil characterization may reduce costs and give predictive and fast results for potential irrigation water risks. However, in addition to salt hazard, risks may be induced by other abundant chemical compounds in the irrigation water/soil that injure the genome without visual effects on plants.

Hazard identification based on the *Vicia*-MCN Test can help to reduce salinity problems by indicating necessary water and soil management. Management options depend on the irrigation water quantity, quality, and frequency, irrigation system, salt leaching, drainage, and the selection of salt-tolerant crop types. Salt-tolerant crops can endure a certain amount of salt without affecting production or quality. Therefore, cultivation of salt-tolerant crops presents a solution of continuous production under different trends and seasonal variation of soil/water salinity. The selection of the crop species depends on the climatic conditions (hot/cool, dry/wet, etc.), the growth stage of the crop, and the varieties of the crop within the same species.

## Figures and Tables

**Table 1:** Physicochemical characterization of water samples. C3 and C4 represent irrigation water classes based on water salinity.

Parameters	Unit	NB	Min	Max	Mean	%CV
C3 class						
pH	Standard	26	7.39	8.05	7.83	2.8
EC	dS m^−1^	26	2.01	2.24	2.10	4.5
Na^+^	me L^−1^	26	6.23	19.86	14.86	35.9
Ca^2+^	me L^−1^	26	3.00	10.00	7.13	38.8
Mg^2+^	me L^−1^	26	1.00	7.50	5.08	46.1
K^+^	me L^−1^	26	0.14	2.14	1.11	43.4
Cl^−^	me L^−1^	26	6.00	20.72	14.19	36.7
SO_4_^2^^−^	me L^−1^	26	1.10	11.63	6.78	61.0
HCO_3_^−^	me L^−1^	26	5.00	9.00	6.72	15.3
SAR		26	3.46	7.65	5.99	20.2
C4 class						
pH	Standard	24	7.01	8.07	7.70	5.00
EC	dS m^−1^	24	3.46	7.00	5.13	23.3
Na^+^	me L^−1^	24	39.00	69.00	51.58	18.4
Ca^2+^	me L^−1^	24	10.00	30.00	20.19	40.2
Mg^2+^	me L^−1^	24	2.50	21.00	11.58	44.5
K^+^	me L^−1^	24	0.17	0.91	0.34	67.4
Cl^−^	me L^−1^	24	36.92	79.00	54.04	24.6
SO_4_^2^^−^	me L^−1^	24	8.90	34.00	23.26	38.7
HCO_3_^−^	me L^−1^	24	4.50	9.00	6.27	22.2
SAR		24	10.57	15.51	13.25	10.6

Cations: Na^+^ = sodium, Ca^2+^ = calcium, Mg^2+^ = magnesium, K^+^ = potassium. Anions: Cl^−^ = chloride, SO_4_^2−^ = sulfate, HCO_3_^−^ = bicarbonates. SAR = sodium adsorption ratio, EC = electrical conductivity, NB = number of samples, Min = minimum, Max = maximum, %CV = coefficient of variation.

**Table 2:** Growth parameters of *V. faba* roots after 48 h of exposure to C3 (EC: 2.01–2.24 dS m^−1^) and C4 (EC: 3.46–7.00 dS m^−1^) water.

Growth Parameter	NB	Min	Max	Mean	%CV	%Variation
RL (cm)						
C3	26	2.48	5.00	3.38	18.82	
C4	24	2.20	3.80	3.00 *	16.30	−11.06
FM (g)						
C3	26	0.23	0.44	0.31	20.77	
C4	24	0.22	0.38	0.28 *	15.35	−10.50
DM (g)						
C3	26	0.020	0.040	0.027	27.10	
C4	24	0.019	0.032	0.024	15.05	−9.60

RL = root length, FM = fresh mass, DM = dry mass, NB = number of samples, Min = minimum, Max = maximum, %CV = coefficient of variation, % of variation = percentage of variation compared to C3. * indicates significant difference between C3 and C4 at *p* < 0.05.

**Table 3:** Pearson correlation between salinity, genotoxicity, and growth parameters. *V. faba* roots were exposed to C3 (EC: 2.01–2.24 dS m^−1^) and C4 water (EC: 3.46–7.00 dS m^−1^) during 48 h.

Parameter	MI		MCN		RL		FM		DM	
	C3	C4	C3	C4	C3	C4	C3	C4	C3	C4
EC	0.163	−0.764 **	0.258	0.56 **	0.244	−0.322	0.197	−0.222	0.199	−0.223
MI	-	-	−0.251	−0.496 *	0.518 **	0.335	−0.341	0.230	−0.344	0.201
MCN	-	-	-	-	−0.075	−0.519 **	−0.031	−0.347	−0.028	−0.362
RL	-	-	-	-	-	-	0.307	0.803 **	0.308	0.705 **
FM	-	-	-	-	-	-	-	-	0.99 **	0.159

EC = electrical conductivity, MI = mitotic index, MCN = micronucleus, RL = root length, FM = fresh mass. n = 26 for C3 and 24 for C4. * indicates significant correlation at *p* < 0.05, ** indicates significant correlation at *p* < 0.01.

**Table 4:** Pearson correlation between ionic elements (cations Na^+^, Ca^2+^, Mg^2+^, and K^+^ and anions Cl^−^, SO_4_^2−^, and HCO_3_^−^), salinity, genotoxicity, and growth parameters. *V. faba* roots were exposed to C3 (EC: 2.01–2.24 dS m^−1^) and C4 (EC: 3.46–7.00 dS m^−1^) water samples during 48 h.

Parameter	Ionic Element						
	Na^+^	Ca^2+^	Mg^2+^	K^+^	Cl^−^	SO_4_^2−^	HCO_3_^−^
C3 Class							
MI	0.382	0.231	0.206	0.045	0.347	0.215	−0.084
MCN	−0.102	−0.001	−0.051	−0.202	−0.030	0.009	−0.147
RL	0.243	0.190	0.177	−0.158	0.302	0.172	−0.280
FM	0.191	0.211	0.209	0.432 *	0.255	0.221	−0.056
DM	0.191	0.212	0.210	0.431 *	0.255	0.222	−0.055
C4 Class							
MI	−0.771 **	−0.465 *	−0.827 **	−0.417 *	−0.805 **	−0.518 **	0.180
MCN	0.592 **	0.481 *	0.466 *	0.915 **	0.606 **	0.456 *	−0.333
RL	−0.290	−0.404	−0.192	−0.514 *	−0.388	−0.203	−0.004
FM	−0.132	−0.426 *	−0.014	−0.305	−0.258	−0.124	−0.232
DM	−0.098	−0.469 *	−0.015	−0.300	−0.219	−0.186	−0.183

Cations: Na^+^ = sodium, Ca^2+^ = calcium, Mg^2+^ = magnesium, K^+^ = potassium. Anions: Cl^−^ = chloride, SO_4_^2−^ = sulfate, HCO_3_^−^ = bicarbonates. MI = mitotic index, MCN = micronucleus, RL= root length, FM = fresh mass, DM = dry mass. n = 26 for C3 and 24 for C4. * indicates significant correlation at *p* < 0.05, ** indicates significant correlation at *p* < 0.01.

**Table 5:** Salinity hazard classes [1].

EC (dS m^−1^)	Salinity Class	Salinity Hazard	Remark on Quality
0.10–0.25	C1	Low	Excellent – It can be used safely for irrigation of most crops.
0.25–0.75	C2	Medium	Good – It can be used if a moderate amount of leaching can occur. Moderate salt tolerant plants can be mostly grown without special practices.
0.75–2.25	C3	High	Doubtful – Even with adequate drainage, special management for salinity control may be required.
>2.25	C4	Very high	Unsuitable—cannot be used for irrigation under ordinary conditions.

EC = electrical conductivity.

**Table 6:** The sodium hazard of water [1].

SAR Values	Class	Sodium Hazard of Water	Remarks and Comments
1–10	S1	Low	Can be used for irrigation on almost all soils with little danger of the soil developing harmful levels of exchangeable sodium.
10–18	S2	Medium	Presents an appreciable sodium hazard in fine textured soils, especially under low leaching conditions. Amendments (such as gypsum) and leaching are needed.
18–26	S3	High	Generally unsuitable for continuous use
>26	S4	Very high	Generally unsuitable for use

SAR = sodium adsorption ratio.

**Table 7:** Guidelines for the interpretation of irrigation water quality according to the joint sodium adsorption ratio and electrical conductivity content [2].

Scheme	Degree of Restriction on Use		
	No Restriction	Slight to Moderate	Severe
	EC of Irrigation Water (dS m^−1^)		
0–3	>0.7	0.7–0.2	<0.2
3–6	>1. 2	1.2–0.3	<0.3
6–12	>1.9	1.9–0.5	<0.5
12–20	>2.9	2.9–1.3	<1.3
20–40	>5.0	5.0–2.9	<2.9

SAR = sodium adsorption ratio, EC = electrical conductivity.

## Data Availability

Data is contained within the article.

## References

[1] United States Salinity Laboratory Staff (1954). Diagnosis and Improvement of Saline and Alkali Soils. Agriculture Handbook (United States Department of Agriculture), No. 60.

[2] Ayers R.S., Wescot D.V. (1985). Water Quality for Agriculture.

[3] Zörb C., Mühling K.H., Kutschera U., Geilfus C.-M. (2015). Salinity Stiffens the Epidermal Cell Walls of Salt-Stressed Maize Leaves: Is the Epidermis Growth-Restricting?. PLoS ONE.

[4] Wang F., Xu Y., Wang S., Shi W., Liu R., Feng G., Song J. (2015). Salinity Affects Production and Salt Tolerance of Dimorphic Seeds of Suaeda Salsa. Plant Physiol. Biochem..

[5] García-Caparrós P., Hasanuzzaman M., Lao M.T., Hasanuzzaman M., Fotopoulos V., Nahar K., Fujita M. (2019). Oxidative Stress and Antioxidant Defense in Plants Under Salinity. Reactive Oxygen, Nitrogen and Sulfur Species in Plants.

[6] Naveed M., Sajid H., Mustafa A., Niamat B., Ahmad Z., Yaseen M., Kamran M., Rafique M., Ahmar S., Chen J.-T. (2020). Alleviation of Salinity-Induced Oxidative Stress, Improvement in Growth, Physiology and Mineral Nutrition of Canola (*Brassica Napus* L.) through Calcium-Fortified Composted Animal Manure. Sustainability.

[7] West G., Inzé D., Beemster G.T.S. (2004). Cell Cycle Modulation in the Response of Thep Primary Root of Arabidopsis to Salt Stress. Plant Physiol..

[8] Wolny E., Skalska A., Braszewska A., Mur L.A.J., Hasterok R. (2021). Defining the Cell Wall, Cell Cycle and Chromatin Landmarks in the Responses of Brachypodium Distachyon to Salinity. Int. J. Mol. Sci..

[9] Qi F., Zhang F. (2020). Cell Cycle Regulation in the Plant Response to Stress. Front. Plant Sci..

[10] Yazdani M., Mahdieh M. (2012). Salinity Induced Apoptosis in Root Meristematic Cells of Rice. Int. J Biosci. Biochem. Bioinforma..

[11] Souguir D., Abd-Alla H.I., Hörmann G., Hachicha M. (2018). Chromosomal and Nuclear Alterations in the Root Tip Cells of *Vicia Faba* Induced by Sodium Chloride. Water Environ. Res..

[12] Hayashi M. (2016). The Micronucleus Test—Most Widely Used In Vivo Genotoxicity Test. Genes Environ..

[13] Iqbal M. (2016). *Vicia Faba* Bioassay for Environmental Toxicity Monitoring: A Review. Chemosphere.

[14] Bonciu E., Firbas P., Fontanetti C.S., Wusheng J., Karaismailoğlu M.C., Liu D., Menicucci F., Pesnya D.S., Popescu A., Romanovsky A.V. (2018). An Evaluation for the Standardization of the *Allium Cepa* Test as Cytotoxicity and Genotoxicity Assay. Caryologia.

[15] Sandoval-Herrera N., Paz Castillo J., Herrera Montalvo L.G., Welch K.C. (2021). Micronucleus Test Reveals Genotoxic Effects in Bats Associated with Agricultural Activity. Environ. Toxicol. Chem..

[16] Fernandes T.C.C., Mazzeo D.E.C., Marin-Morales M.A. (2007). Mechanism of Micronuclei Formation in Polyploidizated Cells of *Allium Cepa* Exposed to Trifluralin Herbicide. Pestic. Biochem. Physiol..

[17] Fenech M., Kirsch-Volders M., Natarajan A.T., Surralles J., Crott J.W., Parry J., Norppa H., Eastmond D.A., Tucker J.D., Thomas P. (2011). Molecular Mechanisms of Micronucleus, Nucleoplasmic Bridge and Nuclear Bud Formation in Mammalian and Human Cells. Mutagenesis.

[18] Russo A., Degrassi F. (2018). Molecular Cytogenetics of the Micronucleus: Still Surprising. Mutat. Res./Genet. Toxicol. Environ. Mutagen..

[19] Sablowski R., Carnier Dornelas M. (2014). Interplay between Cell Growth and Cell Cycle in Plants. J. Exp. Bot..

[20] Geilfus C.-M. (2011). Expansin Expression and Apoplastic PH in Expanding Leaves under NaCl Stress. Ph.D. Thesis.

[21] Julkowska M.M., Testerink C. (2015). Tuning Plant Signaling and Growth to Survive Salt. Trends Plant Sci..

[22] Lindberg H.K., Wang X., Järventaus H., Falck G.C.-M., Norppa H., Fenech M. (2007). Origin of Nuclear Buds and Micronuclei in Normal and Folate-Deprived Human Lymphocytes. Mutat. Res.-Fund. Mol. M..

[23] Ding L., Cao J., Lin W., Chen H., Xiong X., Ao H., Yu M., Lin J., Cui Q. (2020). The Roles of Cyclin-Dependent Kinases in Cell-Cycle Progression and Therapeutic Strategies in Human Breast Cancer. Int. J. Mol. Sci..

[24] Barnum K.J., O’Connell M.J., Noguchi E., Gadaleta M.C. (2014). Cell Cycle Regulation by Checkpoints. Cell Cycle Control.

[25] Ryu H., Cho Y.-G. (2015). Plant Hormones in Salt Stress Tolerance. J. Plant Biol..

[26] Majda M., Robert S. (2018). The Role of Auxin in Cell Wall Expansion. Int. J. Mol. Sci..

[27] Oh M.-H., Honey S.H., Tax F.E. (2020). The Control of Cell Expansion, Cell Division, and Vascular Development by Brassinosteroids: A Historical Perspective. Int. J. Mol. Sci..

[28] Demirkiran A., Marakli S., Temel A., Gozukirmizi N. (2013). Genetic and Epigenetic Effects of Salinity on in Vitro Growth of Barley. Genet. Mol. Biol..

[29] Tuteja N., Mahajan S. (2007). Calcium Signaling Network in Plants: An Overview. Plant Signal. Behav..

[30] Hossain M.S., Dietz K.-J. (2016). Tuning of Redox Regulatory Mechanisms, Reactive Oxygen Species and Redox Homeostasis under Salinity Stress. Front. Plant Sci..

[31] Uz G., Sarikaya A.T. (2016). The Effect of Magnesium on Mitotic Spindle Formation in Schizosaccharomyces Pombe. Genet. Mol. Biol..

[32] Abraham S., Nair R.B. (1989). Production of Mitotic Abnormalities by Magnesium Sulphate in *Vicia faba* L.. Cytologia.

[33] Boyko A., Golubov A., Bilichak A., Kovalchuk I. (2010). Chlorine Ions but Not Sodium Ions Alter Genome Stability of Arabidopsis Thaliana. Plant Cell Physiol..

[34] Unger M.W., Hartwell L.H. (1976). Control of Cell Division in Saccharomyces Cerevisiae by Methionyl-TRNA. Proc. Natl. Acad. Sci. USA.

[35] Tabur S., Oney S. (2009). Effect of Artificial Fertilizers on Mitotic Index and Chromosome Behaviour in *Vicia hybrida* L.. J. Agric. Res..

[36] Barragán V., Leidi E.O., Andrés Z., Rubio L., De Luca A., Fernández J.A., Cubero B., Pardo J.M. (2012). Ion Exchangers NHX1 and NHX2 Mediate Active Potassium Uptake into Vacuoles to Regulate Cell Turgor and Stomatal Function in Arabidopsis. Plant Cell.

[37] Andres Z., Perez-Hormaeche J., Leidi E.O., Schlucking K., Steinhorst L., McLachlan D.H., Schumacher K., Hetherington A.M., Kudla J., Cubero B. (2014). Control of Vacuolar Dynamics and Regulation of Stomatal Aperture by Tonoplast Potassium Uptake. Proc. Natl. Acad. Sci. USA.

[38] Hasanuzzaman M., Bhuyan M., Nahar K., Hossain M., Mahmud J., Hossen M., Masud A., Moumita, Fujita M. (2018). Potassium: A Vital Regulator of Plant Responses and Tolerance to Abiotic Stresses. Agronomy.

[39] Ragel P., Raddatz N., Leidi E.O., Quintero F.J., Pardo J.M. (2019). Regulation of K^+^ Nutrition in Plants. Frontiers in Plant Science.

[40] Assaha D.V.M., Ueda A., Saneoka H., Al-Yahyai R., Yaish M.W. (2017). The Role of Na^+^ and K^+^ Transporters in Salt Stress Adaptation in Glycophytes. Front. Physiol..

[41] Cramer G., Epstein E., Läuchli A. (1989). Na-Ca Interactions in Barley Seedlings: Relationship to Ion Transport and Growth. Plant Cell Environ..

[42] Zhang D.H., Wadswort P., Hepler P.K. (1992). Modulation of Anaphase Spindle Microtubule Structure in Stamen Hair Cells of Tradescantia by Calcium and Related Agents. J. Cell. Sci..

[43] Hepler P.K. (1994). The Role of Calcium in Cell Division. Cell Calcium.

[44] Hepler P.K. (2005). Calcium: A Central Regulator of Plant Growth and Development. Plant Cell.

[45] Hanson J.B. (1984). The Functions of Calcium in Plant Nutrition. Adv. Plant Nutr.

[46] White P.J., Broadley M.R. (2003). Calcium in Plants. Ann. Bot..

[47] Wilkins K.A., Matthus E., Swarbreck S.M., Davies J.M. (2016). Calcium-Mediated Abiotic Stress Signaling in Roots. Front. Plant Sci..

[48] Seifikalhor M., Aliniaeifard S., Shomali A., Azad N., Hassani B., Lastochkina O., Li T. (2019). Calcium Signaling and Salt Tolerance Are Diversely Entwined in Plants. Plant Signal. Behav..

[49] Souguir D., Ferjani E., Ledoigt G., Goupil P. (2008). Exposure of *Vicia Faba* and *Pisum Sativum* to Copper-Induced Genotoxicity. Protoplasma.

